# AKT2在非小细胞肺癌中的表达及预后意义

**DOI:** 10.3779/j.issn.1009-3419.2011.05.03

**Published:** 2011-05-20

**Authors:** 小辉 缪, 勇 宋, 镗烽 吕, 平 展, 艳玲 吕, 冬梅 袁

**Affiliations:** 210002 南京，南京大学医学院临床学院，南京军区南京总医院呼吸内科 Department of Respiratory Medicine, Jinling Hospital, Nanjing University School of Medicine, Nanjing 210002, China

**Keywords:** 肺肿瘤, 人AKT2蛋白, 预后, Lung neoplasms, Human AKT2 protein, Prognosis

## Abstract

**背景与目的:**

AKT2是PI3K信号传导通路中重要因子，AKT2激活导致细胞生长和生存，近年来，许多研究表明AKT2在肿瘤形成、生长及转移中起着重要作用。本研究通过检测肿瘤组织中AKT2的表达水平，旨在研究AKT2在非小细胞肺癌（non-small cell lung cancer, NSCLC）中的表达及其与临床预后的关系。

**方法:**

通过免疫组化方法检测80例NSCLC及10例肺良性病变的组织标本中AKT2蛋白水平。

**结果:**

NSCLC中AKT2表达的阳性率为57.50%（46/80），明显高于肺良性病变组织（1/10, 10.0%）中的表达，具有统计学差异（χ^2^=8.038, *P*=0.006）。AKT2表达与NSCLC患者临床病理特征无明显关系。AKT2表达与患者无进展生存期（χ^2^=12.671, *P*=0.005）及总生存期（χ^2^=9.851, *P*=0.021）有明显关系。

**结论:**

NSCLC中AKT2是患者预后不良的生物学标志。

## Abstract (en)

**Background and objective:**

AKT2 is a critical actor in the PI3K signal transduction pathway. Activation of AKT2 can lead to cell growth and survival. It has been revealed that AKT2 play a central role in tumorigenesis, tumor growth as well as metastasis. The aim of this study is to investigate the association between AKT2 and the clinical outcome of non-small cell lung cancer (NSCLC) patients by detecting its expression levels in the tumor tissue samples.

**Methods:**

We developed an immunohistochemistry (IHC) assay to measure AKT2 protein levels in lung specimens from 80 cases with NSCLC and 10 cases with benign pulmonary disease.

**Results:**

The positive rate of AKT2 was 57.50% (46/80) in NSCLC, which was higher than that in benign pulmonary (10.0%) samples (χ^2^=8.038, *P*=0.006). There was no significant correlation between AKT2 expression and the clinicopathologic profiles. The expression of AKT2 was significantly correlated with the progression free survival (PFS) (χ^2^=12.671, *P*=0.005) and the overall survival (OS) (χ^2^=9.851, *P*=0.021) of patients with NSCLC.

**Conclusion:**

AKT2 may provide a prognostic bio-marker of NSCLC.

AKT2是丝氨酸/苏氨酸蛋白激酶的重要亚型之一，AKT2通过PI3K/AKT细胞信号通路，调控细胞增殖、分化及生存^[1]^。大量证据^[2, 3]^表明，AKT2与肿瘤细胞增殖和转移过程密切相关，在多种肿瘤组织中都存在AKT2蛋白的过度表达。目前AKT2在肺癌中表达及与患者临床病理特征的关系已有相关研究^[4]^，但与患者预后关系的临床报道很少。本研究采用免疫组化方法检测肺癌组织及肺良性病变组织中的AKT2水平，旨在分析AKT2表达及其与肺癌患者的临床特征及预后的关系。

## 1 材料与方法

### 1.1 标本

组织标本由南京胸科医院以及南京军区南京总医院提供，2001年1月-2003年12月收集的95例原发性非小细胞肺癌（non-small cell lung cancer, NSCLC）以及10例对照组肺良性病变（1例肺结核、5例支气管扩张、3例肺大疱、1例炎性假瘤）的手术组织标本，其中15例失访，共80例纳入本研究，术前均未经放化疗。肿瘤组织学TNM分期基于2009版UICC分类法重新分期，包括42例腺癌和38例鳞癌。这项研究得到南京军区南京总医院伦理委员会同意。

### 1.2 方法

采用免疫组织化学染色方法（EnVision），按常规程序脱蜡和复水。通过特定兔抗人AKT2单克隆抗体（Santa Cruz公司）（用PBS液稀释成1:100）孵化过夜。PBS液冲洗后，加入山羊抗兔二抗体（二步法抗兔/鼠通用型免疫组化试剂盒丹麦Dako公司）孵化30 min。PBS液冲洗后，切片用DAB显色剂（北京中杉金桥公司）孵化30 min。用苏木素（北京中杉金桥公司）进行复染。梯度酒精脱水，二甲苯透明，中性树胶封片。AKT2细胞质染色结果按Remmele评分系统^[5]^来计算，计算阳性细胞（显棕黄色）染色密度与百分率。随机选取5个高倍镜视野，每个视野计数100个细胞，计算阳性细胞数占总细胞数的百分比，取5个视野的算术平均值。根据显色细胞的比例计分：5个视野的显色癌细胞百分比的算术平均值< 5%为（-），5%-25%为（+），25%-50%为（++）， > 50%为（+++）。超过5%为阳性， < 25%（--+）为低表达组， > 25%（++-+++）为高表达组。

### 1.3 统计及分析

 80例NSCLC患者从手术日期开始随访，至患者死亡结束，至2009年5月1日仍然生存及无疾病进展者以60个月计算。实验结果采用SPSS 17.0统计软件处理，采用χ^2^检验及*Fisher’s*精确概率检验、*Kaplan-Meire*生存分析，*P* < 0.05为有统计学差异。

## 2 结果

### 2.1 NSCLC及肺良性病变组织的AKT2表达（图 1）

**1:**
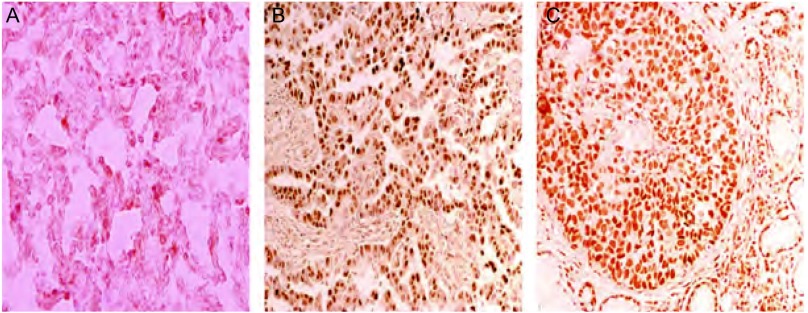
AKT2在非小细胞肺癌以及良性病变组织中的表达（DAB, ×400）。A：AKT2在肺良性病变（肺大疱）组织中阴性表达；B：AKT2在肺腺癌组织中低表达；C：AKT2在肺鳞状细胞癌组织中高表达 Expression of AKT2 in NSCLC and benign lung specimens (DAB, ×400). A: Negative expression of AKT2 in benign lung specimen (pulmonary bullae); B: Low-expression of AKT2 in adenocarcinoma; C: High-expression of AKT2 in squamous cell carcinoma

免疫组化染色显示，AKT2定位于NSCLC和肺良性病变组织细胞质中，表达程度如肺良性病变（肺大疱）组织AKT2阴性表达组（图 1A），肺腺癌AKT2低表达组（图 1B）及肺鳞癌AKT2高表达组（图 1C）。

在80例NSCLC中，AKT2表达阳性为46例（阳性率为57.50%），对照组10例肺良性病变组织中，AKT2表达阳性为1例（阳性率为10.0%），两组存在统计学差异（χ^2^=8.038, *P*=0.006）。

### 2.2 AKT2蛋白在NSCLC组织中的表达及其与临床特征的关系

由表 1可见，AKT2表达与临床特征无明显相关性（*P* < 0.05）。

**1:** AKT2表达与患者临床特征的关系 Relationship between the expression of AKT2 and characteristics of 80 patients with NSCLC

Characteristic	*n*	Expression of AKT2		*X*^2^	*P*
		Negative	Positive		
All patients	80	34	46		
Sex				2.417	0.120
Male	61	23	38		
Female	19	11	8		
Age (year)				1.666	0.197
< 60	38	19	19		
≥ 60	42	15	27		
Smoking				2.035	0.154
Non-smoker	42	21	21		
Smoker	38	13	25		
Size of tumor				3.292	0.070
≤ 3 cm	18	11	7		
> 3 cm	62	23	39		
Histology				0.948	0.330
Squamous cell carcinoma	38	14	24		
Adenocarcinoma	42	20	22		
N stage				0.718	0.397
N0	49	19	30		
N1+N2	31	15	16		
p-TNM stage				0.799	0.372
Ⅰ+Ⅱ	62	28	34		
Ⅲ+Ⅳ	18	6	12		
Grade				0.389	0.533
Well/Moderate	51	23	28		
Poor	29	11	18		

### 2.3 AKT2与NSCLC的无疾病进展生存期（progression-free survival, PFS）和总生存期（overall survival, OS）关系

至随访结束时，生存人数为28例，生存率为35.00%。无疾病进展生存人数为19例。肿瘤中AKT2阳性表达与患者的PFS及OS相关（PFS: χ^2^=12.761, *P*=0.005; OS: χ^2^=9.851, *P*=0.021）（表 2）。

**2:** AKT2表达与患者PFS及OS的关系 Relationship between the expression of AKT2 and PFS and OS of patients with NSCLC

Characteristic	*n*	Expression of AKT2		*X*^2^	*P*
		Negative	Positive		
All patients	80	34	46		
PFS (month)				12.761	0.005
≤ 6	11	1	10		
6-12	23	6	17		
12-24	13	7	6		
> 24	33	20	13		
OS (month)				9.851	0.021
≤ 12	14	3	11		
12-36	27	8	19		
36-60	11	5	6		
> 60	28	18	10		

整体PFS中位时间为17个月（95%CI:10.182-23.818），OS中位时间为3 5个月（9 5%CI : 20.975-49.025）。AKT2表达阴性组中，PFS中位时间为36个月（95%CI: 17.428-54.572），OS中位时间为45个月（95%CI: 38.078-51.040）；AKT2表达阳性组中，PFS中位时间为12个月（95%CI: 9.529-14.417），OS中位时间为22个月（95%CI: 16.308-27.692）。结果提示：AKT2阴性组的PFS和OS中位时间明显长于阳性组。AKT2表达阳性组PFS明显低于阴性组（χ^2^=9.893，*P*=0.002，图 2A），同样两组的OS存在明显差异（χ^2^=9.611，*P*=0.002，图 2B），AKT2阳性组预后明显差于阴性组。

**2:**
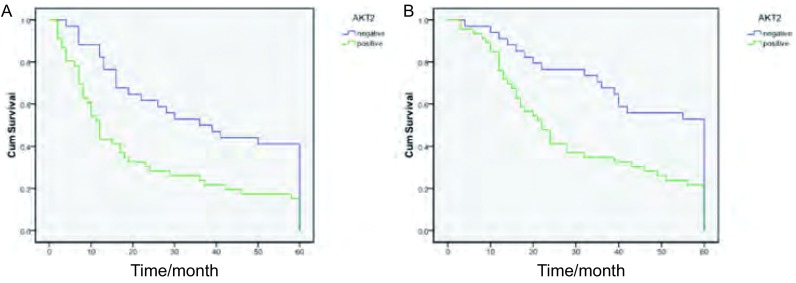
AKT2表达与非小细胞肺癌患者无进展生存期（A）及总生存期（B）的*Kaplan-Meier*生存曲线 *Kaplan-Meier* survival curve of expression of AKT2 in 80 patients with NSCLC. Survival curves (PFS) are stratified by negative and positive AKT2 expression (A); Survival curves (OS) are stratified by negative and positive AKT2 expression (B)

## 3 讨论

肺癌是癌症相关死亡的首要原因，其发病率逐年上升，但目前仍然没有充分有效的治疗手段，5年生存率仅为15%左右。

AKT是一种丝氨酸/苏氨酸激酶，此酶有3种异构体，分别为AKT1、AKT2和AKT3，是PI3K信号传导通路下游因子^[6]^。PI3K家族参与多种信号通路，调节细胞的主要功能。正常情况下，由其活化而产生的类脂产物3, 4-二磷酸磷脂酰肌醇[PI(3, 4)P_2_]和3, 4, 5-三磷酸磷脂酰肌醇[PI(3, 4, 5)P_3_]作为第二信使结合并激活多种细胞内的靶蛋白，形成一个信号级联复合物，最终调节细胞的增殖、分化、存活和迁移等。AKT在PI3K作用下发生磷酸化，磷酸化AKT水平增加可导致细胞生存通路PI3K/AKT的进一步活化，AKT主要介导体内PI3K依赖的细胞粘附、运动、侵袭和转移^[7]^。AKT2是丝氨酸/苏氨酸激酶的重要亚型之一，调控细胞增殖、分化及生存^[1]^。许多研究^[8, 9]^表明人类肿瘤组织中存在*AKT2*基因扩增和mRNA过表达，卵巢癌和胰腺癌中得到证实。*AKT2*已被认为是癌基因，PI3K/AKT成为抗调亡的通路之一^[10]^。AKT2与肿瘤细胞转移和增殖过程密切相关。

我们研究发现，NSCLC组织中AKT2表达阳性率明显高于肺良性病变组织，说明在肺癌组织中存在着AKT2的过度表达。刘红等^[11]^研究报道的结果和本研究结果相似，他们同样运用免疫组化的方法检测NSCLC标本中AKT2表达阳性率增高。AKT2表达与NSCLC临床特征无相关性。

同时，本研究结果中，AKT2表达与NSCLC患者PFS及OS存在密切的相关性，AKT2阳性组的患者预后明显差于阴性组，提示AKT2在NSCLC的进展、转移中起着重要的作用，AKT2表达直接影响NSCLC患者预后，AKT2可作为NSCLC患者预后差的预测因子。

生物学标志物的不断发现，为NSCLC的诊断和治疗提供了重要线索。PI3K/AKT信号通路对于细胞增殖、分化和调亡的调节起着重要的作用，目前对该信号通路在肿瘤侵袭转移中的作用机制已基本明确，以哌立福新（perifosine）为代表的脂类抑制剂是目前抑制AKT的抗肿瘤药物，对肺癌治疗效果的临床Ⅱ期试验正在进行中^[12]^。AKT2将有望成为肺癌治疗的新靶点，通过一些小分子阻制剂对AKT2选择性抑制将成为未来肺癌治疗的新途径^[13]^。相信不久的将来，以PI3K/AKT通路为靶点的小分子药物将有望应用于肺癌的临床治疗中。

总之，我们通过免疫组化的方法检测NSCLC组织及肺良性病变组织中AKT2蛋白的表达情况，分析发现AKT2蛋白的表达与患者临床特征及预后密切相关，对判断NSCLC患者预后具有重要意义。
